# An outline of SARS-CoV-2 pathogenesis and the complement cascade of immune system

**DOI:** 10.1186/s42269-021-00582-2

**Published:** 2021-07-09

**Authors:** Padmalochan Hembram

**Affiliations:** Department of Botany, Berhampur University, Bhanja Bihar, Berhampur, 760007 India

**Keywords:** COVID-19, SARS-CoV-2, PRR, PAMPs, SARS, Acute respiratory syndrome, Angiotensin-converting enzyme 2

## Abstract

**Background:**

Coronavirus disease 19 is a viral infection caused by a novel coronavirus, SARS-CoV-2. It was first notified in Wuhan, China, is now spread into numerous part of the world. Thus, the world needs urgent support and encouragement to develop a vaccine or antiviral treatments to combat the atrocious outbreak.

**Main body of the abstract:**

The origin of this virus is yet unknown; however, rapid transmission from human-to-human “*Anthroponosis”* has widely confirmed. The world is witnessing a continuous hike in SARS-CoV-2 infection. In light of the outbreak of coronavirus disease 19, we have aimed to highlight the basic and vital information about the novel coronavirus. We provide an overview of SARS-CoV-2 transmission, timeline and its pathophysiological properties which would be an aid for the development of therapeutic molecules and antiviral drugs. Immune system plays a crucial role in virus infection in order to control but may have dark side when becomes uncontrollable. The host and SARS-CoV-2 interaction describe how the virus exploits host machinery and how overactive host immune response can cause disease severity also addressed in this review.

**Short conclusion:**

Safe and effective vaccines may be the game-changing tools, but in the near future wearing mask, washing hands at regular intervals, avoiding crowed, maintaining physical distancing and hygienic surrounding, must be good practices to reduce and break the transmission chain. Still, research is ongoing not only on how vaccines protect against disease, but also against infection and transmission.

## Background

In late 2019, many unexplained pneumonia cases observed in Wuhan, China, and the outbreak was confirmed to be caused by a coronavirus (Jin et al. 2020a; Wu et al. 2020b; Liu et al. 2020b). Coronaviruses (CoVs) cause multiple respiratory damages and intestinal infections (Channappanavar and Perlman 2017; Song et al. 2019). In severe cases, they can cause life-threatening pneumonia and bronchiolitis. Individuals with the impaired immune system are at high risk to this infection (Zhang and Liu 2020; Yi et al. 2020). It belongs to the same family of viruses responsible for severe acute respiratory syndrome (SARS) and Middle East respiratory syndrome (MERS) because the genetic sequencing sample of this virus shows about 80% similarity with SARS-CoVs and it shows the same symptoms as SARS and MERS (Ashour et al. 2020).

## Main text

**Fig. 1:**
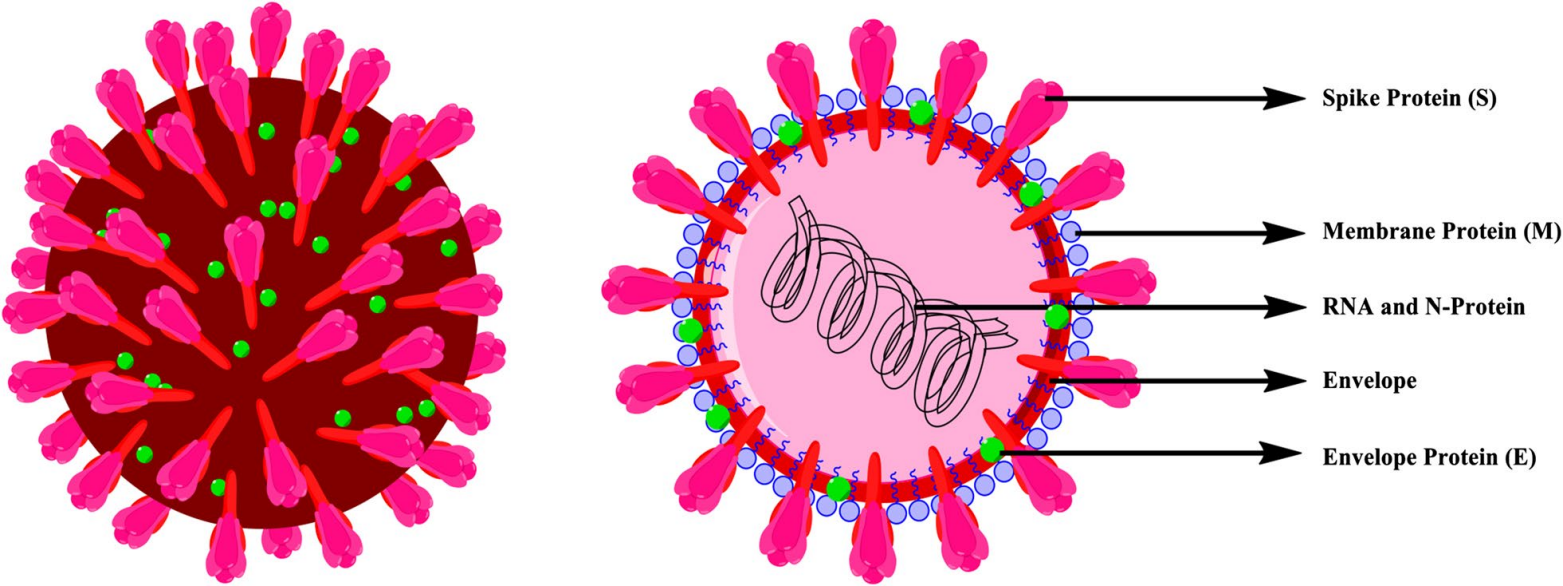
The structure of novel coronavirus SARS-CoV-2

According to the International Committee on Taxonomy of Viruses (ICTV) nomenclature name of new coronavirus (nCoV) fallen as SARS-CoV-2 and the disease as COVID-19 (Jin et al. 2020b; Acter et al. 2020; Nandy 2019; Paudel et al. 2020). Novel coronavirus 2019 is a spherical shaped like other coronaviruses (Wu et al. 2020c; Kim et al. 2020). The virus has a capsid formed by N-protein (nucleocapsid protein) where the viral genome packed inside. The capsid is further covered by envelope where various structural proteins are found (Vennema et al. 1996; Shi et al. 2020; Chaudhary et al. 2020). There are three essential structural proteins found on the envelope surface. These are spike proteins (S), membrane protein (M) and envelope protein (E). Among these, S-protein shows protrusion and mediates virus entry into the host cell and gives the crown-like appearance to the virus shown in Fig. 1.

Coronavirus is positive-sense single-stranded RNA genome viruses that found widely in humans and mammals. In pandemic history, coronavirus was first identified in 2002 of SARS CoV, the causative agent for the severe acute respiratory syndrome (Khan et al. 2020; Pillaiyar et al. 2020; Pal et al. 2020). In 2013, MERS-CoV was identified in Middle Eastern countries for its pathogenicity (Khan et al. 2020; Pillaiyar et al. 2020; Pal et al. 2020). Tremendous research has been carried out on human coronavirus pathogenesis for better understanding and may encourage to discover new potential therapeutic drug or antivirals.

Viral particles exert a complex equilibrium in the host cells by which immune system contributes several responses in the clearance of viruses or to neutralize the infection (Misra et al. 2020). Immune system is always not completely prominent in clearing the virus and viruses evolve various strategies to counteract too. Sometimes, immune system pathways act negatively by invading host cells during acting upon viruses, which is called maladjustive immune system (Rogosnitzky et al. 2020). The innate immune system, an antigen independent response, recruits immune cells mainly involves macrophages, NK cells, dendritic cells. These cells further trigger the production of inflammatory factors which eventually activates cytokines and chemokines. The overproduction of these active inflammatory molecules results cytokine storm by not stopping itself, responsible for attacking and damaging healthy tissues (Rogosnitzky et al. 2020).

There is no clinically approved antiviral drug or vaccines to be used against COVID-19 (Shereen et al. 2020). Over the glove are in great hurry to understand COVID-19 and to find the best to treat it (Malik et al. 2020). However, many things remain unknown, but one thing is clarified that nCoV infection is both highly transmissible and deadly pathogenic (Geng et al. 2020).

## COVID-19 the pandemic: spreading like wildfire

COVID-19, coronavirus disease 2019, is an emerging infectious disease caused by a newly identified coronavirus (Gou et al. 2020). Coronavirus is among the large group of viruses, consisting of RNA as genetic material surrounded by an envelope with spikes of glycoprotein on the surface. People infected by this virus show mild to serious symptoms, from just common cold and fever to discomfort in breathing (Zhu et al. 2020). It may cause pneumonia and kidney failure and lead to death. According to WHO, many people infected with the COVID-19 virus recovered without requiring special treatment (Singhal 2020). Aged people and immunocompromised people having medical issues like diabetes, cardiovascular and cancer are more likely to evolve serious illness (Singhal 2020). There are many cases at present. Both SARS and COVID-19 emerged in China (Wilder-Smith et al. 2020; Porcheddu et al. 2020; Lai et al. 2020). Although COVID-19 virus is not lethal as SARS, the number of infection and death caused by COVID-19 surpasses SARS, now declared as a pandemic (Peeri et al. 2020). The outbreak has spread acrimoniously across many countries in a short space of time since coronavirus has posed a global threat to public health (Brown and Wang 2020; Sen et al. 2020).

The international traveling played a critical role in spreading of the coronavirus from Wuhan of China to different nations across the globe (Driggin et al. 2020). Gathering tribes and communities to the metropolis with very poor screening has sparked opportunities for the amplification of infection. However, this epidemic starts in Wuhan, China, but by the middle of January, several countries reported COVID-19 which signified that it was a globalizing disease and no longer China’s problem alone (Safdar and Yasmin 2020). Moreover, it had become anthroponosis (person-to-person transmitted disease). The COVID-19 virus transmits primarily through droplets of saliva or mucus-like that discharge when an infected person coughs or sneezes. These respiratory fluids or droplets can infect other people by direct contact or can infect those who pick them up by touching infected surfaces and then their face, nose and eyes (Repici et al. 2020). According to the Centre for Disease Control and Prevention (CDC), US, the COVID-19 causing virus seems to be spreading quickly in the community in the affected geographical areas (Cascella et al. 2020). The outbreak was declared a Public Health Emergency of International Concern and world pandemic announced by the World Health Organization (WHO) (Cascella et al. 2020). Till dated on September 12, 2020, the novel coronavirus has infected more than 28,659,610 individuals globally and has killed more than 919,175 individuals worldwide. The number of infections appearing each day what started in Wuhan, China, has now engulfed over 213 countries.

## Coronavirus-host interaction: the basis of disease

**Fig. 2:**
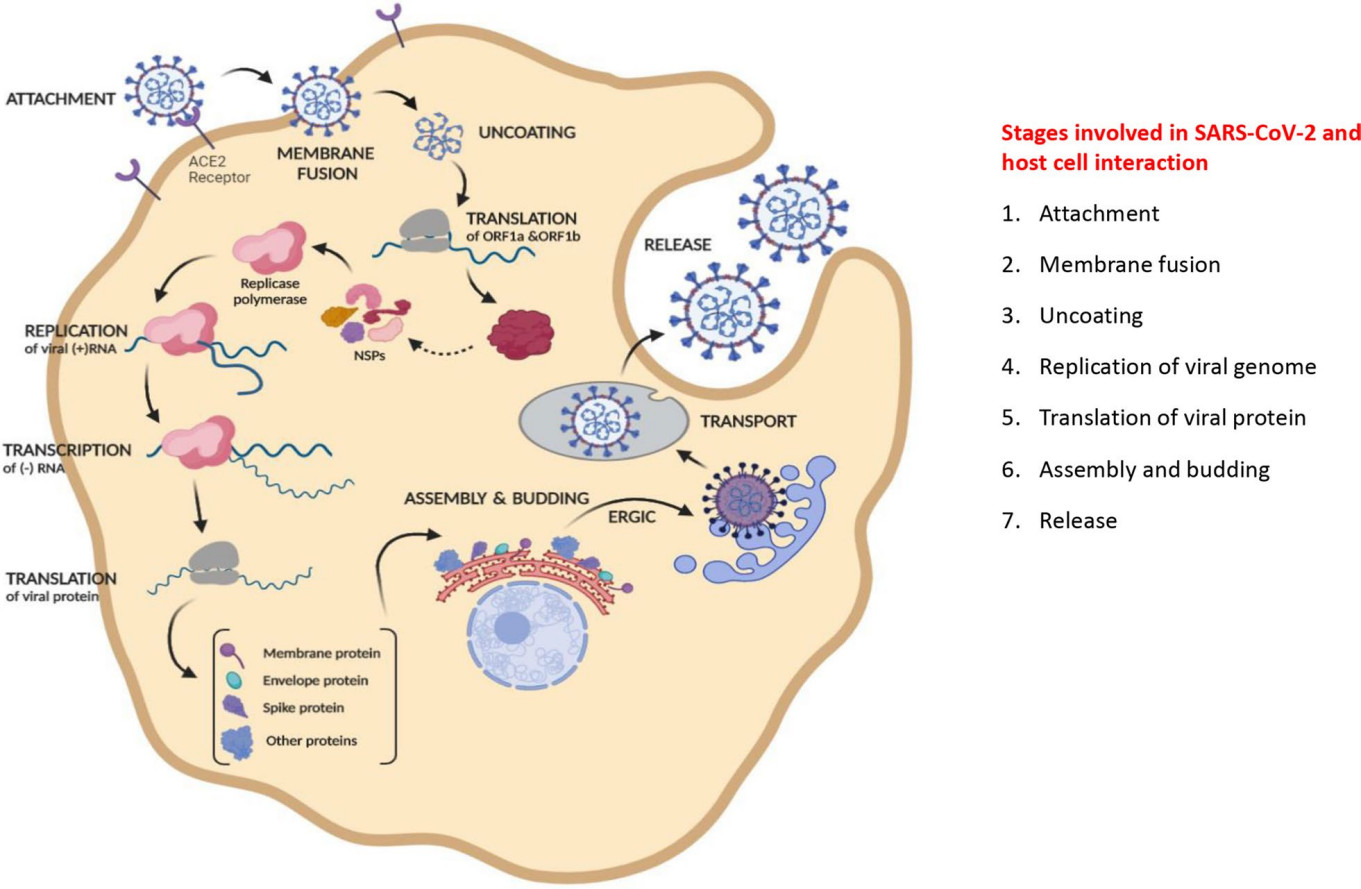
The interaction and lifecycle of coronavirus. Outline of the intracellular obligate parasitic strategy of SARS-CoV-2 to hijack host machinery

Coronaviruses contain specific genes in ORF1 downstream regions that encode necessary proteins such as structural proteins and which helps in their multiplication (Shafiq et al. 2020; Kumar et al. 2020). The glycoprotein spikes present on the outer-surface of CoV play the principal role in attachment and entry into host cells (Shereen et al. 2020; Wan et al. 2020). The entry mechanism of coronavirus depends upon cellular proteins such as Human Airway Trypsin-like proteases (HAT), Cathepsins and Transmembrane Protease Serine 2 (TMPRS2), which help in the splitting of spike proteins and lead further penetration. Coronavirus requires Angiotensin-Converting Enzyme 2 (ACE2) as a key receptor present in human cells. Binding of spike protein to human ACE2 receptor triggers a conformational change that facilitates membrane fusion through the endosomal pathway which leads in the release of viral genetic material into the host cell cytoplasm. Replication of CoV begins with the translation of ORF1a and 1b into polyproteins pp1a and pp1ab. These get cleaved proteolytically that give rise to form nonstructural proteins (NSPs) (Shereen et al. 2020; Wan et al. 2020). The NSPs get assembled and form replicase–polymerase that called replication–transcription complex (RTC) (Shereen et al. 2020; Wan et al. 2020). The replicase–polymerase takes part in the replication of viral genomic RNA (gRNA) and transcription of subgenomic RNA. The subgenomic RNA then undergoes translation to produce structural proteins and other accessory proteins (Shereen et al. 2020; Wan et al. 2020). The fast-track assembly of virions happens with the accumulation of gRNA and viral proteins. The process of assembly completes once budding of nucleocapsid has over followed by transport by secretory vesicles and release out of host cell (Shereen et al. 2020; Wan et al. 2020). The route of the assembly to budding takes place in Endoplasmic reticulum to Golgi intermediate complex (ERGIC). Viral entering and replication provide an initial picture of novel coronavirus pathogenesis, elucidated in Fig. 2.

## Pathogenesis of SARS-CoV-2

The pathophysiology of SARS-CoV-2 infection shows a close resemblance to SARS CoV infection with invasive inflammatory reactions highly associated with the consequences of airway damages (Lai et al. 2020; Yang et al. 2020). Respiratory droplets are the primary mode of SARS-CoV-2 transmission, similar to other respiratory-related coronaviruses (Prompetchara et al. 2020; Jalava 2020). Individuals infected from novel coronavirus usually exhibit chills, sore throat and dry cough, fever, fatigue and breathing difficulties. Repeated shaking with chills, muscle pain and loss of taste sensation are the new symptoms recently put on by Centre of Disease control, USA, sever covid-19 cases headway with acute respiratory distress syndrome (ARDS), characterized by breathing shortness and low blood oxygen level, which directly lead to respiratory failure (Lai et al. 2020). More than about 65 percent of death cases of COVID-19 fatal cases are associated with ARDS. In a pathological finding in National Health Commission (NHC), China, biopsy specimens from lung, liver and heart tissue of an infected individual, showed alveolar damage, hyaline membrane development, specifying ARDS (Guo et al. 2020). There were few shreds of evidence of interstitial mononuclear inflammatory invades while no utmost injury in heart tissue. And the biopsy samples from liver exhibit modest microvesicular steatosis (Malik et al. 2020). Many patients also show a rise in secondary infections by bacteria and fungi. To cope with viral infection as well as secondary infections, the immune system induces an enormous release of cytokines to result in a cytokine storm, which may lead to sepsis kind of diseases (Kumar et al. 2020; Lai et al. 2020). Cytokines are tiny secreted protein molecules or can be called peptide as well released by immune cells that mediate and regulate immunity and inflammation (Tay et al. 2020). Cytokine storm causes unbalanced inflammation that inflicts multi-organ dysfunction or even leads to organ failure, mainly the cardiac system of heart, the hepatic system of the liver and renal system of kidney gets affected (Russell et al. 2020; Prompetchara et al. 2020; Liu et al. 2020b).

The SARS CoV-2 infects cells by entering into the cell by ACE2 (Zhang et al. 2020a; South et al. 2020; Hoffmann et al. 2020). The replication and release of the virus compel the host cell to face pyroptosis (Moccia et al. 2020). Underlying pyroptosis releases pathogen-associated molecular patterns (PAMPs) and damage-associated molecular patterns (DAMPs), which recognized by surrounding cells that triggers the generation of pro-inflammatory protein molecules. These protein molecules attract immune cells to the site of infection that boost more inflammation. The immune cells may include macrophages, monocytes and T-cells (Tay et al. 2020; Coperchini et al. 2020; Neurath 2020). The cells may damage the air-blood barrier by destroying vascular endothelial cells, and airway epithelial cells result in collateral tissue damage. Airway epithelial cells and endothelial cells show high expression of ACE2 receptor, which is utilized by coronavirus to enter inside the cell. Thus, acute sickness is not only because of viral infection but also overactive immune responses (Zhu et al. 2020).

## Immune system response to SARS-CoV-2 attack

Cascades of viral particles enter into the body, hijack cellular machinery, replicate and multiply and start infecting adjoining cells. Viruses has specific signature pattern on their surface which is called antigens that propel host immune system to produce antibodies. The immune system has two arms, innate and adaptive immunity (Zhang et al. 2020c; Connors and Levy 2020; Felsenstein et al. 2020). The innate immunity response is immediately begun with the recognition of pathogen-associated molecular patterns (PAMPs) through pathogen recognition receptors (PRR). PRR includes several kinds of receptors like Toll-like receptors (TLR), Nod-like receptors (NLR), RIG-I-like receptors (RLR), Lectin-like receptors, etc. TLR recognizes lipids, lipoproteins, nucleic acids, proteins of viruses and other microorganism origin. RLR recognizes nucleic acids of RNA viruses, whereas NLR recognizes several components of pathogens and function as inflammasome. Lectin-like receptors is widely expressed in myeloid cells. It takes part in essential signaling pathway that triggers a variety of cellular responses like phagocytosis, chemotaxis of cells and maturation of dendritic cells (Zhang et al. 2020c; Connors and Levy 2020; Felsenstein et al. 2020).

When a virus infect cells, interferon regulatory factors get activated and enhances type-I interferon (IFN) production (Li et al. 2020a). The IFNs promote the downstream JAK-STAT signaling cascade, triggers IFN-stimulating genes (ISGs) expression. The IFNs has a significant role to reduce virus spread. Interferons are protein molecules only, made by our cells in response to viral infections (Li et al. 2020a; Ghias et al. 2020). Too little interferons at early infection and immoderate production at later could contribute to acute or lethal cases. There are three different groups of interferons found in humans: type I, type II and type III (Rajaei and Dabbagh 2020; Noris et al. 2020). All these categories of interferons are produced by immune cells, but type I is produced by infected cells and type III is produced by epithelial cells. Types I and II produce pro-inflammatory active responses, but type III interferes with viral replication and is less inflammatory. Type III interferons also enhance barrier stability to epithelial cells. But viral particles interact with interferons and other antiviral signaling in host cells to halt their functions (Li et al. 2020a).

Pathogen infection begins with the abrupt activation of complement systems in the host (Zhang et al. 2020c; Connors and Levy 2020; Felsenstein et al. 2020). The complement system is an essential component of innate immunity, plays a critical role in pathogen elimination (Rajaei and Dabbagh 2020; Noris et al. 2020). A persistent encounter of toxins and or pathogens takes place in the airway epithelium from the nasal cavity to alveoli. During this the invaders unclog the mucus and breach the epithelium and when it happens, it must contain complement proteins. For instance, C3 is one of abundant protein found in the bronchial epithelial cells of cystic fibrosis patients. C3a and C5a drive pro-inflammatory mediator upregulation associated with airway epithelial cell injury. So, it suggests that complement proteins are found at alveolar epithelium region to fight against pathogens and pathogen deposits (Magro et al. 2020; Noris et al. 2020). Complement system activation promotes the formation of bioactive molecules like C3 and C5 anaphylatoxins (Smole et al. 2020; Gou et al. 2020). Among complement activation molecules, the C5 anaphylatoxin is one of the dominant inflammatory peptides (Smole et al. 2020). C5a performs its function when it binds with its receptor C5aR or C5aL2. C5aR exhibits higher affinity for C5a expresses on both myeloid cells like neutrophils, basophils, monocytes and non-myeloid cells like bronchiolar and alveolar epithelial and endothelial cells in different organs (like lungs and liver) (Smole et al. 2020; Magro et al. 2020; Gou et al. 2020).

C5aR is a G-protein coupled receptor which plays a critical role after C5a binding with it (Magro et al. 2020). A high level of C5a has commonly observed in bronchoalveolar lavage fluid (BALF) and serum of individuals infected from respiratory viruses (To et al. 2020; Magro et al. 2020; Guo et al. 2020). C5a is displayed as a potent chemoattractant and recruits inflammatory cells which involves eosinophils, neutrophils, monocytes, macrophages and T-lymphocytes. C5a also triggers the activation of cytokines and a prolong cytokine release results in “cytokine storm” (Smole et al. 2020; Magro et al. 2020; Gou et al. 2020). Overproduction of C5a is also responsible for the generation of reactive oxygen species (ROS), causes oxidative burst. An experiment carried out in mice infected with influenza virus demonstrates that ROS released from respiratory, immune and inflammatory cells prompts lung damage. C5a modulates innate immunity by inducing immune cells to generate cytokines, including IL-12, tumor necrosis factor-α (TNF) and macrophage inflammatory proteins-1α (MIP). It also modulates adaptive immune cells to generate IL-1β, IL-6 and IL-8. In an experiment, anaphylatoxin complement system deficient mice infected by SARS-CoV exhibited less respiratory dysfunction and lower the level of cytokines and chemokines in both lung and serum which signifies complement system (C3a, C5a) blockade may alleviate the inflammatory lung complication of COVID-19 (Jiang et al. 2018).

**Fig. 3:**
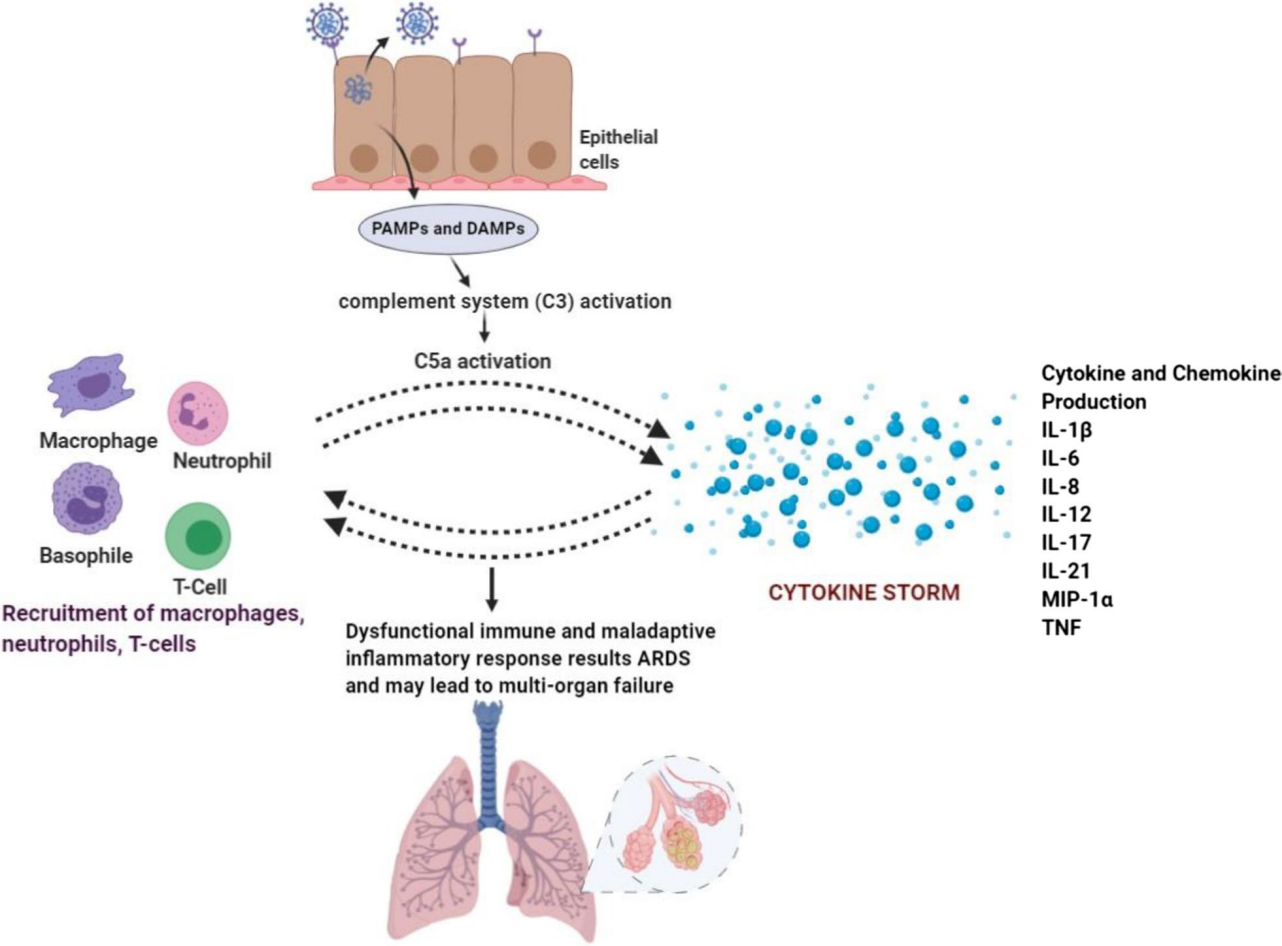
Dysfunctional host immune response against SARS-CoV-2 can cause the disease more severity

Cytokine storm mediated by C5a had observed in influenza virus infected individuals (Hu et al. 2016). C5a downregulates TLR4 (Toll-Like Receptor) to induce IL-12 family cytokine expression that can play a critical role in amplifying acute lung injury (ALI) and ARDS (Xiang and Fan 2010; Hashimoto et al. 2004; Puneet et al. 2005). Thus, elevated levels of pro-inflammatory cytokines and chemokines produced in response to novel coronavirus might be a reason for causing ALI, ARDS and multi-organ failure, illustrated in Fig. 3. Therefore, inhibiting cytokine and chemokine production can have a protective effect against maladaptive inflammatory response during coronavirus infection.

## The antiviral treatment approach for SARS-CoV-2: an urgent need

Formulating potential vaccine, particularly for the virus, is a time enduring process. Antibodies from recovered patients are already tested and used, which can also act potential treatment for the infection. (Ludwig and Wagner 2007; Daughton 2020; Lega et al. 2020). Antibodies are immunoglobulins that our immune system produces to neutralize the pathogens. Convalescent plasma therapy (CPT), typical adaptive immunotherapy, has been exercised in the prevention and treatment of ancient pandemics such as SARS, MERS, 2009 H1N1. In 2014, WHO recommended the CPT for Ebola virus disease treatment (Van Bavel et al. 2020; Bansal et al. 2020). The concept of CPT is to utilize plasma, the opaque liquid part of the blood from a recovered patient who has developed humoral immunity that able to remove or neutralize the virus. It is used to treat during the occurrence of severe adverse conditions, but the clinical benefits and peril of this therapy for COVID-19 still uncertain (Bansal et al. 2020). Therefore, it will be worthwhile to examine its efficacy and safety measures.

To bring this pandemic to an end, many research teams across the world developed vaccines that protect from SAR-CoV-2, the virus that causes COVID-19. As of now several vaccines are available, among which national regulatory authority have granted emergency use authorization for sixteen vaccines, and currently vaccine has been administered worldwide. Some of the recommended vaccines are CoronaVac, Covaxin, Sputnik V, Oxford–AstraZeneca, Convidecia, Pfizer–BioNTech, Moderna, etc. However, some drug molecules believed to be effective, have reviewed in Table 1. Antiretroviral treatments like Lopinavir and Ritonavir are also under clinical trials. The overactive and maladjustive inflammatory response is thought to targeted with anti-inflammatory drug molecules such as AMY-101, Ravulizumab and Ifenprodil which are registered under investigation (Zhai et al. 2020; Lythgoe and Middleton 2020). All current authorized and recommended vaccines are categorized under four major types, mRNA vaccines, inactivated virus vaccines, adenovirus vector vaccines and protein subunit vaccines. mRNA vaccines are those in which a copy of natural mRNA used that can gear up strong immune response. Inactivated virus vaccines consisting of destroyed disease producing viral particle, whereas adenovirus vector vaccines are the vaccines that use pathogen genes in order to stimulate good immune response, genes used are generally antigens coding surface protein of the coronavirus. The protein subunit vaccines contain one or more antigens (fragments of pathogens). The humanity has often relied on vaccination technology in the past in order to bring down the infection and deaths causing by infection. The vaccines basically stimulate immune system and build T-lymphocytes and B-lymphocytes which will recognize the virus and remember how to protect and fight with virus causing COIVD-19 in future infection.

**Table 1:** Illustrate a few potential treatments for COVID-19 approved for human clinical trials

Vaccine/drug molecule	Clinical trials	Description
ADZ1222 (ChAdO × 1 nCoV-19)	NCT0432606	Attenuated Adenovirus exhibit SARS-CoV-2 spike protein in its surface (Zhang et al. 2020b; Cai et al. 2020)
Ad5-nCoV	NCT04313127, NCT04341389, NCT04398147	Replication-impaired Adenovirus integrated with a full length nCoV-19 spike protein (Shi et al. 2020; Lythgoe and Middleton 2020)
INO-4800	NCT04336410	Based on plasmid DNA encoded with antigens found in nCoV-19 (Le et al. 2020)
AMY-101	NCT04395456	Pro-inflammatory complement system C3 blockade agent and hope with C5a inactivation (Zhou et al. 2019; Mastaglio et al. 2020)
Ravulizumab	NCT04369469, NCT04390464	Monoclonal antibody-based treatment that halts the activation of inflammatory component system C5 (Zhou et al. 2019; Lee et al. 2019; Kulasekararaj et al. 2019)
Baricitinib	NCT04321993, NCT04345289, NCT04393051	It is aimed to inhibit JAK/STAT signaling pathway to reduce cytokine production (Richardson et al. 2020)
Ifenprodil	NCT04382924	It is an N-methyl-D-Aspartate inhibitor targeted to alleviate T-cell cytokine production (Allen et al. 2020)
Interferon-α	NCT04320238	It is a cytokine mainly produced by the immune system to fight against the army of a virus (Cao et al. 2020)
Convalescent plasma	NCT04321421, NCT04338360, NCT04397757	Plasma-based immunotherapy, obtained from COVID-19 recovered patient, which contain specific antibiotics, enzymes and essential proteins that may be protective against nCoV-19 (Liu et al. 2020a; Chen et al. 2020; Shen et al. 2020; Duan et al. 2020; Bloch et al. 2020)
Ribavirin	NCT04356677, NCT04276688	It is a nucleoside analog antiviral molecule induce mutation in the viral genome that can be lethal (Li et al. 2020b; Guo et al. 2020; Dong et al. 2020; Khalili et al. 2020; Hung et al. 2020)
Remdesivir	NCT04292730, NCT04280705, NCT04373044	A nucleoside analog antiviral drug that interferes with SARS-CoV-2 replication (Devaux et al. 2020; Wu et al. 2020a; Wang et al. 2020; Grein et al. 2020)
Ritonavir	NCT04321993, NCT04350684, NCT04366245	It is a protease inhibitor that can be very effective by inhibiting viral proteases and host proteases that degrade antivirals (Huang et al. 2020; Dariya and Nagaraju 2020; Lim et al. 2020; Cao et al. 2020)
Lopinavir	NCT04303299, NCT04255017, NCT04376814	It is an anti-retroviral drug that works by binding with viral proteases (Shetty et al. 2020; Chandel et al. 2020; Cao et al. 2020)
Artemisinin	NCT04387240, NCT043872040	It is also used to treat malaria but believed to have antiviral properties (SEHAILIA 2020; Smit et al. 2020)

## Conclusions

The world has experienced coronavirus epidemic first in 2002 of SARS-CoV, then 2012 of MERS-CoV and currently experiencing SARS-CoV-2. No doubt, this outbreak is highly warning by inducing serious illness, human-to-human transmission and rapid rate of spreading infection that declared as a pandemic. SARS-CoV-2 enters into the lung cells by binding with ACE2, along with cells of other organs such as liver, gastrointestinal organ and kidney are also vulnerable due to expression of ACE2 receptors. Coronavirus disease 2019 causes severe sickness, whereas our immune system is fueling new tension on the response that may lead to ARDS and even multi-organ failure. Controlling the complement system, which is responsible for the maladaptive inflammatory response, can be an essential factor in developing potential therapeutic drug candidates. The present scenario of pandemic 2019 is acutely worsening and needs immediate action which can limp back to normalcy. Therefore, the purpose of developing COVID-19 treatments should focus on controlling the maladjustive immune system along with virus clearance. There are many pharmaceutical industries in the race to scale up new therapeutics that can interfere at any stage of the coronavirus infection cycle.

Vaccines being developed and its inoculation has begun, but deep research is needed to upgrade a better understanding of coronavirus and their interaction with the host cell. Openness and sharing of scrutinized data are paramount. Increasing our understanding of coronavirus can expand opportunities not only in rational design and development of therapeutics but also to target new coronavirus in future emergence.

## Data Availability

Not applicable.

## Abbreviations

ACE2: Angiotensin-converting enzyme 2
ALI: Acute lung injury
ARDS: Acute respiratory distress syndrome
BALF: Bronchoalveolar lavage fluid
CDC: Centre of disease control
COVID-19: Coronavirus disease 2019
CPT: Covalescent plasma therapy
DAMPs: Damage-associated molecular patterns
ERGIC: Endoplasmic reticulum to Golgi intermediate complex
FDA: Food and drug administration
HAT: Human airway trypsin-like protease
ICTV: International committee on taxonomy of virus
IFN: Interferons
ISGs: Interferon stimulating genes
MERS: Middle East respiratory syndrome
MIP: Macrophages inflammatory proteins
nCoV: Novel corona virus
NK: Natural killer cells
NLR: Nod-like receptor
NSPs: Nonstructural proteins
ORF: Open reading frame
PAMPs: Pathogen-associated molecular patterns
PRR: Pathogen recognition receptor
ROS: Reactive oxygen species
RTC: Replication–transcription complex
SARS: Severe acute respiratory syndrome
TLR: Toll-like receptor
TNF: Tumor necrosis factor
WHO: World Health Organization
